# International Survey Exploring Rider-Perceived Sidedness of the Horse

**DOI:** 10.3390/ani15131956

**Published:** 2025-07-02

**Authors:** Russell MacKechnie-Guire, Hilary Clayton, Anna Byström, David Marlin, Kevin Haussler, Selma Latif, Nadine Blum, Sarah S. le Jeune, Mary Wanless, Agneta Egenvall

**Affiliations:** 1 Equine Department, Hartpury University, Gloucester GL19 3BE, UK; 2 Department of Large Animal Clinical Sciences, College of Veterinary Medicine, Michigan State University, 736 Wilson Road, East Lansing, MI 48824, USA; claytonh@msu.edu; 3 Department of Applied Animal Science and Welfare, Faculty of Veterinary Medicine and Animal Science, Swedish University of Agricultural Sciences, P.O. Box 7024, SE-750 07 Uppsala, Sweden; anna.bystrom@slu.se; 4 Animalweb Ltd., The Granary, Hermitage Court, Hermitage Lane, Maidstone, Kent ME16 9NT, UK; dm@davidmarlin.co.uk; 5 College of Veterinary Medicine, Lincoln Memorial University, Harrogate, TN 37752, USA; kevin.haussler@lmunet.edu; 6 Pferdepraxis Vetcheck GmbH, Wierezwil-Rüberi 273, 3255 Rapperswil, Switzerland; selmalatif@vetcheck.ch; 7 Tierklinik Lüsche GmbH, Essener Straße 39, Lüsche (GER), 49456 Bakum, Germany; nblum@tierklinik-luesche.de; 8 Department of Surgical and Radiological Sciences, University of California, Davis, CA 95616, USA; sslejeune@ucdavis.edu; 9 Independent Researcher, 1 Burtons Bank, Church Westcote, Chipping Norton OX7 6SE, UK; mewanless@hotmail.com; 10 Department of Clinical Sciences, Faculty of Veterinary Medicine and Animal Science, Swedish University of Agricultural Sciences, P.O. Box 7054, SE-750 07 Uppsala, Sweden; agneta.egenvall@slu.se

**Keywords:** asymmetry, handedness, lameness, crookedness, straightness, laterality

## Abstract

Laterality or sidedness in the horse is commonly addressed as part of the horse’s training. An international online survey regarding horse and rider sidedness and asymmetry was conducted, with 2304 useable responses. Confidence intervals (95%) were used to evaluate if some response options were chosen more frequently. Discounting answers of ‘not sure’ or ‘not applicable’, the right fore hoof was more often perceived to be more upright (22%) than the left (15%), and the left shoulder was more prominent (24%) more often than the right (17%), whilst most respondents reported no difference. Both lunging and bending was perceived to be most difficult to the right (48% and 52%) than to the left (33% and 42%), and more horses fell in on the right circle (lunging 40%, riding 38%) than on the left (31% and 33%). Horses commonly tracked their hindquarters to the left in walk, trot and left canter. Correspondence analysis showed that responses indicating horse side bias and relative symmetry, respectively, were associated across several questions, suggesting consistent individual patterns. While the mechanisms underpinning sidedness remain unclear, this study provides insights into horse owners’ perceptions of laterality and related matters.

## Simple Summary

Equestrians often perceive horses to show a preference for one side of their body over the other, which can affect their movement and behaviour during training. To explore this, an international survey was conducted with over 2300 horse riders and owners, focusing on their observations of their horses’ movements and sidedness. Respondents reported that horses more frequently had their manes falling to the right, their right front hooves were more upright, and their left shoulders were more prominent. Horses were reported to struggle more with movements and exercises, such as bending or lunging, on the right rein. Many horses were reported to move their hindquarters to the left when walking, trotting, or cantering to the left. While clear patterns of left or right preference were noted, a substantial number of horses were perceived as mostly symmetrical. These findings support that horses have natural asymmetries, but the underlying reasons remain unclear. Understanding these tendencies can help horse owners and trainers develop more balanced training techniques, for the benefit of horse welfare and performance.

## 1. Introduction

Laterality manifests as asymmetrical use of corresponding left and right body parts, e.g., feet or hands, and is influenced by asymmetric use of the cerebral cortices. Laterality can be divided into different areas: cerebral, indicating how one hemisphere of the brain is dominant when processing different functions compared to the other hemisphere; sensory, a preference to use one sensory organ (eye, ear or nose) when reacting to a stimulus. Horses that use one limb, or side of their body, when performing certain tasks have been described as having a motor laterality. A side preference may lead to asymmetrical body development which may result in a horse described as having body asymmetry. Whilst these areas are often attributed to laterality, it remains unknown if these observations are truly signs of laterality or are acquired [1].

Previous works have addressed both side preferences in individual horses [2,3,4,5,6] and perceptions about general side preferences in the equine population [7]. The latter is commonly described as the horse having a weak and/or a stiff side. The rider perceives this as a more or less pronounced stiffness and resistance to the action of their rein and/or leg on one side or when moving in one direction, compared to the other direction [8]. Equestrian texts suggest that a majority of horses show a bias to the same side, being stiffer/stronger on the right side of the body [9]. An expression of this, for example, is that the horse is easier to lunge when travelling to the left. It is currently unclear to what extent riders are aware of laterality as a scientific term, or its potential relationship with the horse’s ridden performance.

It is generally accepted amongst trainers that it is desirable for the horse to use the left and right sides of the body equally. A horse that shows left-right kinematic or postural asymmetries during training is described as being ‘crooked’. This is in contrast to a symmetrical horse that is described as ‘straight’. Straightness forms part of the “Scale of Training”, a training system that is followed by many riders and trainers [10]. Straightness is characterised by kinematic symmetry, reflecting symmetrical muscle strength and activation. Equestrians recognise straightness by criteria such as the hind hooves following the tracks of the front hooves, which is indicative of axial body alignment, the hind limbs producing symmetrical propulsive forces, and the musculature of the forelimb providing equal elevation. Riders identify a lack of straightness as a habitual lateral bending or axial rotation to one side, the hindquarters deviating laterally relative to the forehand, or a tendency to fall towards one shoulder when turning. Straightness is a training goal in many competitive equestrian sports [11], and in those points may be deducted if the horse’s axial body alignment is not maintained. Most equine professionals, as well as physiotherapists and equine veterinarians, would agree that continuing to work a horse in a crooked posture is detrimental to its locomotor health [12]. However, it has been proposed that enforcing straightness may be stressful and possibly counterproductive to the horse’s psychological and physical balance [13]. This highlights the need to determine the mechanical and functional mechanisms underlying asymmetry and side preference in horses.

The term laterality has been applied to explain the horse’s preference for use of one limb [14], ear [15], or eye [16,17] over the other; however, it is unknown if these manifestations reflect true laterality or acquired habits [1]. Horse owners have reported perceived left-right differences, sidedness and preferences [18,19,20], although defining their origin from a laterality perspective remains challenging [1]. Owners of 65 owner-sound horses responded to questions on laterality, and horses were measured for vertical movement asymmetry of the head and croup when trotting in a straight line. In the same study, 40 horses also underwent a forelimb protraction preference test [18]. No convincing associations were found between these three evaluations [18]. The same group examined 123 high-performing riding horses in a similar manner and found no association between movement asymmetry in trot and perceived laterality [19]. Trainer-evaluated sidedness in 30 horses from one centre was summarised as follows: for 83% of the horses, the left side was considered the weak side, and for 17%, the right side [8]. In 15 unmounted horses, associations were found between motion patterns on left and right circles and trainer-perceived sidedness [20]. There is therefore some scientific evidence of laterality in horses, whilst there is a paucity of evidence for an association between laterality and sidedness as perceived by equestrians [18,19]. In particular, few attempts have been made to objectively measure those expressions of sidedness that equestrians perceive to be common in horses [7].

In contrast to the indistinct findings for horses, it is common knowledge that the human population is right-handed to an overwhelming degree. A recent meta-analytic study found that only 10.6% of all humans studied are left-handed if using a stringent definition of left-handedness, and 18.6% if using more-lenient criteria for non-right handedness [21]. A meta-analysis of footedness found that 23.7% met the criteria for non-right footedness [22].

Compared to cerebral and sensory laterality, equestrians likely have experienced sidedness and body asymmetry in some form when training, riding and caring for horses; therefore, these two formed the foundations of the study. The objectives of this study were to (1) describe and compare the rider’s perceptions of the extent to which the horses they train show structural and functional characteristics frequently described as expressions of laterality/sidedness; (2) describe self-assessed laterality amongst riders; and (3) explore riders’ familiarity with the term laterality, using an online survey. It was hypothesised that the responses would collectively indicate left and right asymmetry or side preference in individual horses in unequal proportions, suggesting population bias.

## 2. Materials and Methods

### 2.1. Survey Design

A survey was developed addressing horse and rider asymmetry, side preference and rider laterality, targeting participants over 18 years of age, who owned or cared for a horse. The survey was developed in English and translated into French, German and Spanish. (Supplement S1 contains the English questionnaire in full as given, including the general instructions provided at the start). A draft of the survey was pilot tested by six equestrian professionals to evaluate usability and clarity. The survey was then edited based on their feedback.

The survey contained 32 questions (Supplement S1); however, each question could contain one or several sub-questions. Each sub-question had two or more response options. For most questions the participant was asked to select the best fitting answer, but a few questions were multiple choice. Respondents were able to save their responses and complete the survey at a later date. This allowed participants to evaluate their horse in the stable before answering. The intended design was that each question was answered before the respondent could move to the next one. However, for questions 18 (shoulder falling in/out while lunging), 22 (different inside/outside rein contact on circles), 24 (shoulder falling in/out on circle when ridden), and 25 (hindquarter deviation), an error allowed participants to proceed to the second sub-question without answering the first.

Questions about respondent demographics included country of residence, age, gender, type of equestrian activities the respondent participated in, and training level. Demographic questions for the horses were age, gender, breed, colour, use(s), and level of training. For breed and colour, a free text answer could be given if a matching option was lacking. For their own equestrian activities and horse use, respondents could choose one or more of 18 options, including ‘other’. For horse and rider training level, respectively, respondents were asked to select beginner, novice, intermediate or advanced for each activity.

Questions specifically addressing rider laterality were “which hand do you write with”, “which arm is uppermost when you fold your arms”, “which leg would you use to kick a ball”, and “which foot would you raise to stand on a chair”. For each question, respondents could answer left, right or ambidextrous. Questions also addressed which side the horse was handled from and which side the horse was mounted from.

Questions about horse side preferences were generally based on equestrian terms and concepts used to describe (non-lameness) asymmetries (e.g., do the horse’s shoulders fall to the inside/outside when ridden or lunged on a circle to the left/right?), as well as asymmetries commonly reported during training (e.g., are both fore and both hind steps of equal length in piaffe or does one limb take a longer step?). For the majority of questions in this category, options ‘not sure’ and/or ‘not applicable’ were also available.

At the end of the survey, there was a “yes/no” question asking if the respondent was familiar with the concept of laterality, followed by a free-text question where they could comment on the subject or the survey in general. Findings from the latter have not been included in this manuscript.

### 2.2. Survey Distribution

The survey was created in Survey Monkey^TM^ (version 3.0.1, Momentive Inc., San Mateo, CA, USA). The survey link was shared via social media (Facebook^®^, Twitter^®^, Linked In^®^) primarily by the authors and advertised by industry regulatory bodies and in national and international (United Kingdom, Germany and North America) press. Respondents were anonymous; however, if respondents chose to participate in a draw with the possibility of winning one of three £100 Amazon vouchers, they were asked to provide their email address at the end of the survey. The survey was live for 102 days, from November 2022 to February 2023.

### 2.3. Data Handling and Processing

Data were downloaded via the SurveyMonkey website (2023) as Excel files (version 2016, Microsoft, Redmond, Washington, USA) and processed in Matlab (version R2020a, Mathworks, Natick, MA, USA) using custom-written scripts.

Data from all responses (number (n) = 4370) were scrutinised for early dropouts and fictitious data such as bot responses or respondents only motivated by the incentive (vouchers). During screening a handful of suspicious patterns were identified. Based on those and the aim of the study, the following criteria for exclusion were applied.

(a) Implausible responses. In 708 responses all equestrian activities and all horse disciplines had been ticked; these were deemed scripted/automated responses. In 10 additional responses, the free-text questions had irrelevant answers, thereby also deemed scripted/fictitious.

(b) Minimal response time. It was deemed unlikely that a human paying any attention would complete the questionnaire in six minutes or less. 1174 responses were completed in ≤6 minutes and therefore deemed automated.

(c) Minimal relevant information: 1350 respondents failed to answer the questions about horse conformational and movement asymmetries (mane, the front hooves, shoulder and hindquarter conformation, tail carriage, lunging, handling, mounting, bending, canter lead, response to rider’s legs), saddle slip and rider upper body posture.

In summary, a total of 2066 of the 4370 answers met one or more of the exclusion criteria. Hence data from 2304 questionnaires were retained and used in the analysis. Prior to statistical analysis, answers to whether the mane fell to the left or the right side for upper, middle and lower parts of the neck were combined to if the mane was overall balanced (equal number of parts falling left and right or falling to both sides) or if it fell predominantly to the left or to the right side. For colour and breed there were many free text answers, and similar answers combined, e.g., spelling variations in the same words or answers with the similar meaning but in different languages. Some answers were difficult to translate consistently across the four languages.

### 2.4. Statistical Analysis

Data analysis was made using SAS (version 9.4, SAS Institute Inc., Cary, NC, USA). Continuous variables (horse age, response time duration) questions were reported as medians, minima and maxima or 5th and 95th percentiles. All other variables were categorical. For evaluating whether all response alternatives were equally common or not, 95% binomial confidence intervals [23] were calculated. When two 95% confidence intervals just barely overlap this generally equates to approximately *p* = 0.05, and non-overlapping confidence intervals indicate that two percentages are significantly different. In these analyses, missing, ‘not applicable’ and ‘not sure’ responses were ignored to facilitate interpretation.

Multiple correspondence analysis (SAS-procedure proc corresp) was undertaken to study patterns in the data. Multiple correspondence analysis returns ‘inertia’, which translates to the variance in principal component analysis (PCA). The principal inertias of each dimension loosely correspond to eigenvalues in a PCA and represent the proportion of variation explained by each dimension [24]. Dimensions correspond to principal components (eigenvectors) in a PCA and are similarly sorted by their inertia value. In a successful analysis, the majority of the total inertia will be explained by the first 2–4 dimensions. Variable coordinate values for each dimension, which correspond to loadings in a PCA, can identify groups of variables with strong covariance. Variables, in our case response options, that share large values for a dimension, describe a common response pattern. In contrast, response options with large values but with opposite signs suggests those are seldom seen together in the data.

Two correspondence analysis models were made. In this analysis the variables were response options which were tagged with their corresponding question. First, a smaller model (model A) containing response options for questions regarding rider laterality, horse handling, mounting, direction which the rider’s upper body collapses and familiarity with the term laterality was made. Second, a larger model (model B) with the same variables as model A plus all equine asymmetry-related response options (including recent lameness, lame on a left diagonal, lame on a right diagonal, lame left laterally and lame right laterally). The definition for whether a horse was lame on the left diagonal was if the horse had lameness on both or either of the left fore and the right hind. The opposite was true for lameness on the right diagonal. The definition for whether a horse had left- sided lameness was if the horse had lameness on both or either the left fore and left hind and the opposite was true for the right side. For each dimension, variables with the largest positive and largest negative contributions, respectively, were examined.

## 3. Results

### 3.1. General Results

For the 2304 answers included in the analysis, the median response time was 11 minutes (5th percentile 7 min and 95th percentile 60 min). The longest response duration was 18 days, indicating that some respondents revisited the questionnaire (questions of conformation encouraged participants to check their horse). The majority of respondents, 1963 (85%) answered the English version, whilst 153 (7%) answered the French version, 170 (7%) the German version, and 18 (1%) the Spanish version. Some questionnaires were incomplete. There was a fairly steady reduction in number of responses from the first to the last question, no specific question caused respondents to leave the survey.

### 3.2. Rider Demographics

Of the 2304 answers, 2177 were from females (94%), 106 from males, (5%), 5 others (0.2%) and 16 (0.7%) who chose ‘prefer not to say’. Respondents reported their age as 18–24 years (n = 148, 6%), 25–34 years (n = 416, 18%), 35–44 years (n = 493, 21%), 45–54 years (n = 494, 21%), 55–64 years (n = 472, 20%) and 65 years or above (n = 265, 12%). Age was not stated in 16 cases (0.7%). Of the 50 countries represented (Supplement S2: Table S1), most respondents were living in the USA (n = 781, 34%), followed by the United Kingdom (n = 740, 32%) and Germany (n = 137, 6%). The most common activities to participate in, at any level, were dressage (n = 1999, 87%), showjumping (n = 1372, 60%), pleasure riding (n = 1340, 58%) and eventing (n = 1025, 45%). Experience level was registered for each discipline. For example, for dressage experience, levels were 13% beginner (n = 266), 34% novice (n = 679), 35% intermediate (n = 689), and 18% advanced (n = 365). In general, for all disciplines (n = 9467 answers in total), experience levels were 21% beginner, 32% novice, 30% intermediate, and 18% advanced. Note that riders could categorise themselves differently for different disciplines/activities, e.g., advanced in dressage and novice in eventing, accordingly the number of answers for experience level was much higher than the number of respondents.

### 3.3. Rider Laterality

Of all respondents, 86% stated they write with their right hand, 47% would have their right arm uppermost when folding arms, 85% would kick a ball with their right foot and 56% would put the right foot up first when stepping on a chair (Table 1). For all four questions, few regarded themselves as ambidextrous/ambipedal (3–11%). Most commonly the horse was handled either always, or mostly from the left (together representing 59% of the answers). Specifically, it was most common to handle the horse mostly from the left (44%), followed by handling from both sides (37%). Most respondents answered they always mount from the left side (64%), followed by mostly from the left side (24%). Regarding collapsing one side while riding, 360 respondents indicated they were ‘not sure’. Of the remaining 1944, most respondents stated they did not collapse their upper body (38%), followed by collapsing to the inside on the left rein (30%). Of the 2299 respondents who answered the final question about laterality, 85% reported they were familiar with the term laterality.

### 3.4. Horse Demographics

Median age of the 2304 horses was 12 years, ranging from 1 to 49 years (49 years is likely a response mistake). There were 1349 geldings (59%), 855 intact mares (37%), 90 stallions (4%) and 10 ovariectomised mares (0.4%). The most common activities to participate in, at any level, were dressage (n = 1958, 85%), showjumping (n = 1150, 50%), pleasure riding (n = 1077, 47%) and eventing (n = 689, 30%). Experience level was registered for each discipline. For example, for dressage experience levels were 25% (n = 484) beginner, 38% novice (n = 739), 28% intermediate (n = 543), and 10% advanced (n = 192). In general, for all disciplines (n = 7319 answers in total), experience levels for horses were 29% beginner, 33% novice, 26% intermediate, and 12% advanced.

Most of the 2304 horses were crossbreeds (27.1%), followed by Warmbloods (26.3%), English Thoroughbreds (10.4%), Quarter horses (5.5%), Iberian horses (5.4%), Irish sport horses (3.0%), Arabian thoroughbreds (2.8%), Welsh ponies from sections A-D (2.2%), cobs (2.1%), draught horses (1.9%), Connemara ponies (1.6%) and Icelandic horses (1.4%). Most respondents chose one of the predefined options, but there were quite a few free text answers. The latter were grouped with similar breeds where applicable (Supplement S2: Table S2). The most common coat colours were bay (37.1%), chestnut (18.5%), grey (14.0%) and black (11.5%). For other colours see Supplement S2: Table S3.

### 3.5. Horse Sidedness

Responses to the 15 questions related to horse asymmetry/side preference are summarised in Table 2. Percentages reported do not include response options ‘not sure’ and ‘not applicable’ (a corresponding table with those alternatives included can be found in Supplement S2: Table S4). Based on non-overlapping confidence intervals, a number of significant differences in right versus left side bias were found. The mane more often fell to the right side (65%) than the left (27%), while equally on both sides was least common (7%). Most respondents answered their horse had symmetrical front hooves. When this was not the case, it was more common that the right hoof was more upright and the left flatter (22%), than vice versa (15%). In most horses both shoulders were equally prominent (59%); however, if one shoulder was more prominent it was more often the left (24%) than the right (17%). Hindquarter muscle development and tail carriage were also perceived as symmetric in most horses (79% and 73%, respectively), with no significant difference in the frequency of left versus right asymmetry.

**Figure 1:**
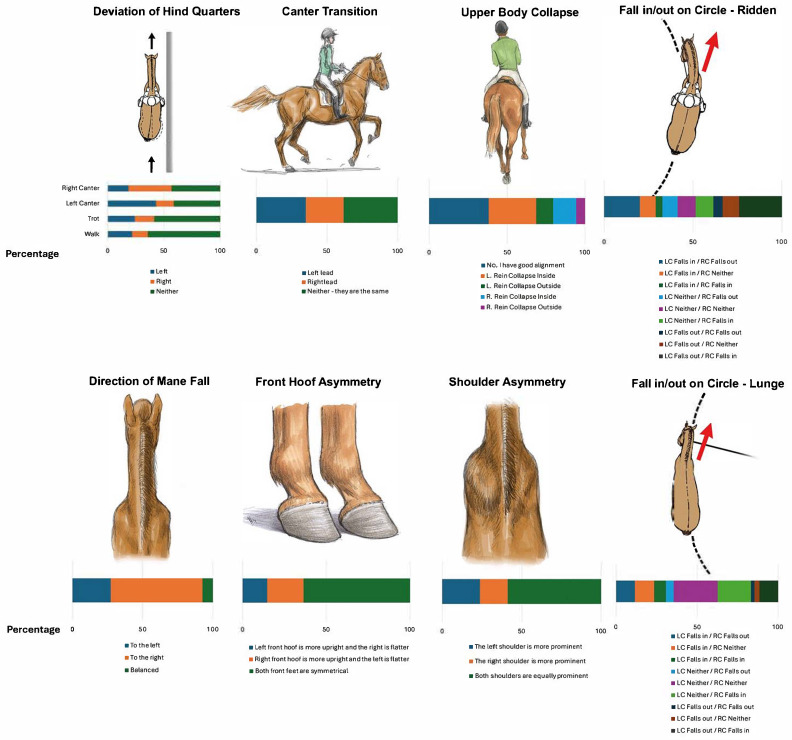
Illustrating the direction (left/right) or neither response for: deviation of the hind quarters when in walk, trot and canter; ease of canter transition; upper body collapse; fall in/out when riding; direction of mane fall; front hoof asymmetry; shoulder asymmetry; and fall in/out when lunging. When two 95% confidence intervals just barely overlap, this generally equates to approximately or just above *p* = 0.05. LC = left circle, RC = right circle, L = left, R = right. The illustrated percentages can also be found in Table 1 and Table 2.

In walk and trot, in most horse’s the hindquarters tracked straight, whilst in canter it was equally common that the hindquarters tracked straight and deviated to the leading side, left side in left canter and right side in right canter. If the hindquarters deviated in walk or trot, it was most often to the left (Table 2, Figure 1), and deviation was somewhat more common in trot than walk. Horses picked up the canter equally easily on both leads (38%) or more easily on the left lead (35%) compared with the right lead (27%). There was no statistical difference in ease of moving away from the left or right leg aid, but either alternative was more common than the horse being equally easy to both sides. Sixty-eight percent of the responses stated that the saddle did not slip. When the saddle slipped, it slipped more often to the right (17%) than to the left (12%). There was no significant difference whether piaffe steps were longer for left or right limbs; however, there were much fewer responses than for other questions (Table 2).

Horses were more often either slightly or substantially easier to bend to the left than to the right (from Table 2, easier to the left: 834 slightly and 333 significantly of 2253 answers = 52%, 95% CI 49.7–53.9; easier to the right: 659 slightly and 276 significantly = 42% 95% CI 39.5–43.6). Regarding rein contact (Table 3), equal contact on both reins in both directions was reported most often (17%). Of the asymmetric patterns, more inside rein contact going to the left and more outside rein contact going to the right, i.e., more contact on the left rein in both directions, was numerically most frequent (14%) but the confidence interval overlapped with several other asymmetric patterns (Table 3).

More horses were reported to fall inwards on circles to the right when lunged (40%) or ridden (38%), than on left circles (lunged 31%, ridden 33%, Table 3). Lunging was more often reported to be easier on the left rein (48% of the horses) compared with easier on the right rein (33% of the horses) (Table 2, Figure 1). When lunging, 52% of horses did not fall in or out on the left circle and on the right circle the same was true for 42%. Considering both directions the most common pattern was that the horse did not fall in or out in either direction during lunging (27%). When ridden, more horses fell outwards on the left circle (39%) than on the right circle (34%). Considering both directions whilst ridden, the most common combination was the horse fell out on the left circle and in on the right circle (24%), i.e., the horse drifted towards its right side in both directions. Second most common was the opposite pattern, falling out on the left circle and falling in on the right circle (20%). In contrast to lunging, only 10% of horses did not fall in or out in either direction when ridden.

### 3.6. Lameness

Of the 2304 horses, 1525 horses (66%) were stated to be sound with the no recent history of lameness while 779 horses (34%) had been lame during the previous 12 months, of which 217 (9%) had been diagnosed with multiple limb lameness. Localisation of the lameness was left fore in 245 cases (11%), right fore in 237 (10%), left hind in 218 (9%), and right hind in 247 (11%). In 237 horses, pathology at locations other than limbs was reported to have caused/contributed to the lameness. These were: neck 72 (3%); back 118 (5%); ‘other’ 47 (2%).

### 3.7. Multiple Correspondence Analysis

In the multiple correspondence analysis, data for all response options were used, including ‘not sure’ and ‘not applicable’. Each response option was included as a separate variable, but tagged with an acronym indicating which question it belongs to, for easier interpretation. Only data from questionnaires with complete answers for the analysed questions were used. Based on relative decrease in fraction of the total inertia (variance explained) by each dimension, results for 3 dimensions are reported for model A, and 5 dimensions for model B. For each dimension the variables with the largest positive and negative loadings, respectively, are shown, 5 + 5 variables for model A (Table 4) and 10 + 10 variables for model B (Table 5).

#### 3.7.1. Model A–Rider Variables

The first three principal inertias represent 7.8%, 7.7% and 6.2% of the total inertia (variation), summing to 21.8% of the total 100% (note that these relatively low percentages are common for multiple correspondence analysis).

In dimension 1, the four variables with the highest positive loading are all from the category ‘ambidextrous’. The lowest negative loadings have a smaller absolute value than the positive loadings (Table 4), i.e., closer to the grand mean, as the data are zero-centred in the analysis process. The negative loadings also do not seem to represent any consistent pattern. This indicates that ambidextrous persons stand out from the majority.

In dimension 2, the three variables with the highest positive loading all relate to left side bias, while the variables with the lowest negative loadings all represent right side bias. The negative loadings are again small, indicating that the left-handed stand out from the majority of respondents, who were mostly right-handed.

In dimension 3 the four variables with the highest positive loadings all relate to handling and mounting from the right side, either mostly or always. The lowest negative loadings are again smaller but represent ambidexterity or doing things from both sides. Note that for this dimension all variables with high positive loadings had a few observations, e.g., in 1% answers respondent state they always mount from the right side.

#### 3.7.2. Model B–Horse Sidedness Variables

The first five principal inertias represent 4.6%, 3.9%, 3.0%, 2.6% and 2.5% of the total inertia, summing to 16.7% of the total 100%. For dimension 1 (Table 5) the variables with highest positive loadings are all from the category ‘not sure’, while the highest negative loadings are all response options which indicate relative symmetry, that left and right sides were equal. This suggests that respondents who chose either of these options tended to stick to the same response across several questions.

For dimension 2, the 10 variables with highest positive loadings possibly relate to strength differences between left and right sides, including both functional symmetries and a history of lameness, while the lowest negative loadings all represent answers ‘not sure” or ‘not applicable’. Possibly this dimension separates between, on the negative end, horses mainly used for trail riding or similar and more seldom worked in the arena, contrasted to the higher-level dressage horses, where unequal strength might be more obvious and/or concerning to the rider, given the higher demands, and in some cases may reflect past or present (subclinical) injuries.

Dimension 3 appears to mainly separate between horses with versus without a history of lameness, if looking at the four variables with the highest positive loadings. However, the remaining variables with high loadings for this dimension do not seem to describe any clear pattern. Again, the negative loadings have a smaller magnitude than the positive loadings.

For dimension 4 the negative loadings represent not sure (−1, −2, −4, −5, −6, −9), or no side preference (−3, −7, −8, −10). The positive loadings have a somewhat higher magnitude but represent a mix of variables not easily interpretable.

For dimension 5, the highest loadings represent a right biased horse with a tendency to fall into the circle on the left rein, that is also handled from the right side. The variables include mounting and handling from the right side (1,3); lunging more easily on the right rein (4]) falling in during lunging and riding on the left rein (5,6); falling out when lunged on the right rein (8); being easier to bend to the right (2,10) and picking up the right lead canter more easily (7). Eight of the negative loadings represent the opposite pattern (a left biassed horse), e.g., easier to bend the left (−1, −7); falling to the inside when lunged or ridden on the right rein (−2, −3); easier to lunge on the left rein (−4); falls out on the circle when lunged and ridden on the left circle (−8, −10), and has more rein contact on the inside rein in the right direction (−6) as well as easier to pick up left lead canter (−5).

## 4. Discussion

The present study describes the results from a questionnaire directed towards and answered by a large number of participants representing diverse geographic locations, equestrian disciplines, and experience levels. For the questions addressing horse asymmetry or side preference, many respondents selected options indicating they perceived their horses as relatively symmetrical, suggesting that not all horses show an obvious side bias or that if it is present, owners are unaware. This was especially true for questions about the horse’s conformation, whilst during riding or lunging it was more common that riders perceived differences between left and right directions. Based on non-overlapping confidence intervals, patterns indicative of population-level side bias were found; for several questions estimated proportions of horses showing left versus right side asymmetries were not equal.

### 4.1. Horse Sidedness

When equestrians discuss sidedness, one key aspect talked about is the horse’s ease of “bending” around the inside leg, i.e., bending throughout the neck and back and ease of turning in either direction. In this study 52% of horses were perceived as bending more easily to the left, compared with 42% bending more easily to the right. These figures also show that it was much more common that the horse was perceived as easier to bend to one side compared to the other, than the horse being equally easy to bend to either side. In dressage, horses are trained to maintain a vertical posture and bend to the inside when turning rather than leaning into the curve to generate the necessary centripetal force [25].

Anatomical constraints for spinal bending are largely due to the orientation of the facet joints. The cervical spine has oblique facet joints oriented at about 45° to the horizontal that allow a large range of lateral motion. From T2 to T16, the horizontal facets allow some lateral bending and axial rotation but in the lumbar region the vertically oriented facets preclude bending motion [26,27,28]. In contrast to dressage, in other disciplines such as barrel racing and showjumping, leaning to the inside is necessary for fast, short radius turns but horses are still expected to perform equally on the left and right sides.

Aside from ease of bending, other possible indicators of laterality or sidedness discussed by equestrians include body alignment [6] and the direction in which the shoulders (pectoral girdle) deviate on the circle [12]. When striving to achieve straightness, the goal is that the horse’s hind hooves should follow the tracks of the front hooves [11]. In the current study, most horses, about 60%, were perceived to accomplish this for straight-line walk and trot, but in canter the hindquarters tend to track to the side, more commonly the inside, (40% of horses). While there was no evidence of a population bias in canter, left deviation was relatively more common in walk and trot, and the correspondence analysis further showed that right hindquarter deviation in walk and trot was linked with right side deviation in left lead canter. These findings suggest asymmetric patterns are present both on population and individual level. Interestingly, 27% of horses did not fall in or out with their shoulders in either direction when lunged compared with only 10% of horses when ridden. This may reflect that horses move more symmetrically when not having to manage the weight of the rider but could also reflect the rider’s greater focus on the shoulders when riding versus watching the horse on the lunge. More horses drifted towards the right forelimb, i.e., fell in on the right circle and out on the left circle. This finding agrees with data from bilaterally handled 9-month-old foals (N = 29) and 2-year-old horses (N = 17). When trotting around a round pen to the right, 9 foals (31%) cut across the circle (derailed) during all 20 circles and 3 of these foals derailed on a single circle to the left. In the 2-year-olds, 10/19 (53%) derailed on all 20 circles to the right and one horse derailed on every circle to the left. These findings suggest an increase in motor laterality with age [29]. In equestrian texts, this pattern along with the horse being easier to bend to the left is described as ‘left hollow’ [6,8] and ‘stiff right’. Counting horses that fell inwards in right direction and fell out or not at all in left direction, 34% of horses showed this pattern while ridden and 33% when lunged.

Many horses have more difficulty making a transition to canter on one lead. However, easier canter departs in either lead has rarely been described as a prominent sidedness indicator by equestrian masters, at least in their written legacy. The results of this questionnaire indicated that 70% of horses picked up either lead equally easily or found the left lead easier. Further, the results of the correspondence analysis showed that easier canter departs to the right were associated with the horse also being easier to bend to the right and lunge to the right, and tending to fall outwards in right direction and inward in left direction, all of which suggest the right side to be the horse’s ‘hollow side’.

Drifting towards one side and not tracking straight are likely to involve asymmetric limb loading and muscle use which, in turn, could be reflected in the horse’s body. Symmetrical shoulders were reported in 59% of horses, with the left shoulder being more prominent in a majority of the asymmetrical horses which is similar to other reports [30]. Similarly to shoulder asymmetry, 64% of horses had symmetrical front hooves and, in those that were asymmetrical, the flatter hoof was the left fore in 22% and the right fore in 15% of horses. In foals bred for high-level dressage, a preference (or acquired behaviour) to systematically graze with one forelimb protracted and the contralateral forelimb retracted, has been reported by 27 weeks of age; this adopted grazing posture was associated with the development of uneven front hoof angles [5] which can influence vertical and longitudinal ground reaction forces [31].

A high percentage of respondents reported that their saddle remained straight when riding. However, for those who reported saddle slip, the saddle more often displaced to the right than to the left side. A saddle which slips to the right is reportedly only apparent on the left rein [32]. A relationship between saddle slip and hind limb lameness has been reported, with the saddle slipping towards the lame or more lame hind limb [33], although saddle slip can also occur in non-lame horses [32,34]. A saddle that slips to the outside on turns and circles alters the rider-horse-saddle interaction [32,35]. The rider’s seat follows the movements of the saddle and slips towards the outside and, as a compensation strategy, the riders lean their upper body to the inside. This give the impression of being concave on the side toward which the upper body is leaning [32,35]. Three hundred and sixty respondents indicated that they were ‘not sure’ if they collapsed through their upper body to the inside or outside. In respondents who were aware of collapsing their upper body it was reported to occur considerably more commonly when riding on the left rein than the right rein. When the rider leans the pelvis and trunk or collapses their upper body to one side, their centre of mass moves toward that side resulting in an increase in force on the saddle on that side [30].

### 4.2. Rider Laterality

Whilst the rider’s upper body position may be influenced by the saddle-horse interaction, the rider’s own laterality is worthy of consideration as a contributing factor. Each rider has their own asymmetries, as well as asymmetric equestrian practices, e.g., always handling the horse from the left side, and the effect on the horse should not be overlooked. In a group of riders, when asked to stand on a measuring device, they had greater load through their right limb, and when asked to symmetrically sit on a static platform they had more weight bearing through their left hemipelvis [36]. It is not known what effect this has on the horse. When asymmetry was induced by shortening one stirrup, differences in the horse’s limb loading contralateral to the shortened stirrup were reported [37] but it is not known how the horse responds to rider asymmetry in the longer term, for example, by developing a compensatory strategy to mitigate the effects. Under such circumstances, the horse’s response to rider asymmetry may be perceived as laterality, and interplay between rider and horse asymmetry is worthy of further investigation.

Using writing hand to define handedness, the percentage left-handed respondents, 12%, is larger than that reported in most previous studies, and also higher compared to a meta-analysis estimate, using strict criteria for left handedness [21]. The meta-analysis found that men had a 2.09% higher frequency of left-handedness than females. Our sample only included 5% males, in light of this a slightly lower percentage would therefore be expected. Three percent (n = 65) stated they would write with both hands, indicating ambidexterity. This is more than was found in a study investigating mixed handedness in children (1%) [38]. Amongst respondents in our study that reported a difference in rein tension, it was most common to perceive higher tension on the left rein on both left and right circles. It seems reasonable to expect that there may be a relationship between rider handedness and forces transmitted to the rein; however, when riding a simulator, higher rein forces were reported for the right rein regardless of rider-reported handedness and the pattern for left-handed riders did not mirror that for right-handed riders [39]. In a group of high-level, right-handed dressage riders, left and right rein forces were similar regardless of direction of travel or gait, but the variation between strides was greater for the dominant (right) hand compared with the non-dominant hand [40].

In a meta-analysis of 164 studies (including 143,135 persons), the combined estimate of non-right footedness was 23.70% (95% CI 21.40–25.90) [22]. From our ball-kick question, the estimate was lower; only 15% of the respondents were non-right footed. However, based on chair-stepping 44% of respondents were non-right footed. A reactivity test in which riders standing with their eyes closed were gently pushed forward, showed a relationship between the leg that initiated movement during the test and saddle force asymmetry in the standing horse [30], which may be related to rider asymmetry/preferences. Riders use their legs and seat to transmit subtle weight aids and cues to the horse, and it would be worth investigating how rider footedness affects horse-rider interactions. The correspondence analysis did not suggest any direct associations between horse sidedness and rider laterality, either handed- or footedness, which may be expected given that both are independent variables. However, it could still be worth investigating this using biomechanical measurements, which could detect more subtle associations that are difficult for riders to pick up on using self-assessment. Further, many questions were yes/no or left/right type questions, which might have been too crude for detecting associations.

A large proportion of respondents indicated that they were familiar with the term laterality (85%). From how the question was asked, it is unclear if those respondents just knew of the word or if they had knowledge of the concept. Correspondence analysis showed that responding ‘not sure’ to one question was associated with similar responses to other questions. This possibly suggests that some respondents were less aware of, or more unsure about, sidedness expressions in their horses. There are several reasons why a respondent might select ‘not sure’, such as different levels of knowledge or self-confidence. These personality traits may confound associations [41].

### 4.3. Multiple Correspondence Analysis

Based on the assumption that side preferences or laterality would be consistent across several situations, it was assumed that answers to several of the survey questions would be highly collinear. Further, it is not really possible to select a single outcome variable among horse asymmetry variables, neither based on data nor prior knowledge. The sheer number of response options required dimensionality reduction. Analysing associations between pairs of variables, using something like chi-square analysis, was not feasible. Correspondence analysis is a dimensionality reduction technique which is useful when a categorical dataset contains many potentially collinear variables and no clear outcome variable, as in our case. Multiple correspondence analysis may loosely be considered a multiple chi-square method, enabling grouping of collinear variables that replicate each other. Results are often displayed as scatter plots, but this was not possible here due to the large number of variables. A caveat with correspondence analysis is that there are no clear criteria for how many dimensions to consider, or how high loadings should be for a variable to be considered important for a particular dimension. Many response options with high loadings in the correspondence analysis were selected by relatively high numbers of respondents. But there were also more unusual response options with high loadings (Table 4 and Table 5). For example, mounting mostly or always from the right side was selected by only 27 and 26 respondents, respectively, is considered unusual for an equestrian. Still, these response options showed up with high loadings, demonstrating that both common and rare answers can influence the results of correspondence analysis. This is similar to a PCA which also has some tendency to pick up outliers.

### 4.4. Limitations

First and foremost, asking riders about their perception of sidedness of their horses will not yield unbiased information about horse sidedness, rather the information will be subjective and coloured by riders’ experiences and peers. Further, it cannot be assumed that all riders who completed the survey had equal ability to assess sidedness: many riders had limited experience and considered themselves beginners or novice, thereby also likely to have ridden a low number of horses regularly, both of which would limit their ability to identify horse sidedness and differentiate it from rider-induced asymmetries. It is not known whether the indicators and characteristics included in the survey truly represent laterality or reflect acquired behaviours, preferences and/or motion patterns [1], more to what extent the expressions of these traits were influenced by the skill of the rider. The use of an incentive to encourage participation may have enticed some respondents to complete the survey with the sole purpose of having a chance to win the reward and possibly also not submitting honest data. We removed many answers, but it remains possible that a few plausible yet fictitious answers were included in the final dataset. The criteria-based process we used likely retained a few fictitious responses and excluded some correct data. However, these errors were judged to be minor (<1%) of the final dataset. Another issue was that while respondents were encouraged to fill out the questionnaire once for each horse they had in their care, it was not possible, because of confidentiality reasons, to identify horses belonging to the same respondent. Based on the time and effort it would take to complete the questionnaire multiple times, and that relatively few riders ride multiple horses regularly, the number of repeated responses by the same rider is likely negligible in the dataset as a whole. However, given that the respondents were offered the option to remain anonymous, there was no way for us to track users across entries if they chose to fill out the survey several times. If possible, rider as a clustering effect should have been accounted for in the statistical analysis, but it was deemed highly unlikely that doing so would have dramatically altered the results reported in this study.

## 5. Conclusions

This survey has shown that indicators of sidedness are common in horses and their riders. However, response options indicating relative symmetry were also chosen frequently and showed associations across several questions. The findings suggest that further information should be sought regarding how equine sidedness translates to neuromotor and biomechanical behaviours, and how these interact with rider asymmetries. In order to accomplish this, we suggest that large-scale, longitudinal biomechanical measurements of large numbers of horses and riders should be gathered, then analysed using techniques such as machine learning and multivariate analyses.

## Figures and Tables

**Table 1:** Counts (n) and percentages with 95% confidence intervals (95% CI) for responses to questions related to rider laterality. These questions, except for the last two (questions 29 and 31), were answered by all 2304 respondents. Total n is shown in the rightmost column.

Question/Response Option	n	Percent	Total/95% CI
Which hand do you write with? (WRITINGHAND)			2304
Ambidextrous (I use both; It varies)	65	2.8	(2.2, 3.6)
Left	267	11.6	(10.3, 13.0)
Right	1972	85.6	(84.1, 87.0)
Please fold your arms-Which arm is uppermost? (ARMFOLD)			2304
Ambidextrous (I use both; It varies)	112	4.9	(4.0, 5.8)
Left	1111	48.2	(46.2, 50.3)
Right	1081	46.9	(44.9, 49.0)
If you were asked to kick a ball foot would you use? (BALLKICK)			2304
Ambidextrous/Ambipedal (I use both; It varies)	169	7.3	(6.3, 8.5)
Left	177	7.7	(6.6, 8.8)
Right	1958	85.0	(83.5, 86.4)
If you were asked to stand on a chair which leg would you place on the chair first? (CHAIRSTEP)			2304
Ambidextrous/Ambipedal (I use both; It varies)	256	11.1	(9.9, 12.5)
Left	749	32.5	(30.6, 34.5)
Right	1299	56.4	(54.3, 58.4)
From which side do you handle your horse? (HANDLING)			2304
Always from the Left Side	354	15.4	(13.9, 16.9)
Mostly from the Left Side	1015	44.1	(42.0, 46.1)
Both Sides	860	37.3	(35.3, 39.3)
Mostly from the Right Side	59	2.6	(2.0, 3.3)
Always from the Right Side	16	0.7	(0.4, 1.1)
From which side do you mount your horse? (MOUNTING)			2304
Always from the Left Side	1464	63.5	(61.5, 65.5)
Mostly from the Left Side	543	23.6	(21.8, 25.4)
Both Sides	241	10.5	(9.2, 11.8)
Mostly from the Right Side	28	1.2	(0.8, 1.8)
Always from the Right Side	28	1.2	(0.8, 1.8)
When riding your horse does your upper body collapse to the inside or outside? (BODYCOLLAPSE)			1944
No, I have good alignment	745	38.3	(36.2, 40.5)
When I ride on the Left rein, I collapse to the inside	589	30.3	(28.3, 32.4)
When I ride on the Left rein, I collapse to the outside	211	10.9	(9.5, 12.3)
When I ride on the Right rein, I collapse to the inside	289	14.9	(13.3, 16.5)
When I ride on the Right rein, I collapse to the outside	110	5.7	(4.7, 6.8)
Are you familiar with the term “laterality”? (LATERALITY)			2299
No	353	15.4	(13.9, 16.9)
Yes	1946	84.6	(83.1, 86.1)

**Table 2:** Counts (n) and percentages with 95% confidence intervals (95% CI) for responses to questions related to horse sidedness. There were 2304 respondents, lower total n (single number in rightmost column) indicates that some participants responded ‘not sure’, ‘not applicable’ or not at all.

Question/Response Option	n	Percent	Total/95% CI
Which side does your horse’s mane fall on? (MANE_FALL)			2304
Balanced	170	7.4	(6.3, 8.5)
To the left	626	27.2	(25.4, 29.0)
To the right	1508	65.5	(63.5, 67.4)
Are your horse’s front feet asymmetrical-one more upright, with a higher heel than the other? (FRONT_HOOVES)			2108
Both front feet are symmetrical	1342	63.7	(61.6, 65.7)
Left front hoof is more upright and the right is flatter	309	14.7	(13.2, 16.2)
Right front hoof is more upright and the left is flatter	457	21.7	(19.9, 23.5)
When viewed from behind and high up, does your horse have one shoulder that is more prominent than the other? (SHOULDER_SYMMETRY)			1983
Both shoulders are equally prominent	1168	58.9	(56.7, 61.1)
The left shoulder is more prominent	472	23.8	(21.9, 25.7)
The right shoulder is more prominent	343	17.3	(15.7, 19.0)
When viewed from behind, does your horse have more muscle development on one hindquarter when compared with the other (e.g., bigger, higher, more muscled)? (HINDQUARTER_MUSCLE)			2131
Both appear equal	1680	78.8	(77.0, 80.6)
The left hind has increased muscle development	245	11.5	(10.2, 12.9)
The right hind has increased muscle development	206	9.7	(8.4, 11.0)
When moving in a straight line, does your horse carry the tail to one side? (TAIL_CARRIAGE)			2188
The tail carriage changes from left to right or neutral	288	13.2	(11.8, 14.7)
The tail is usually carried centrally	1592	72.8	(70.8, 74.6)
The tail is usually carried to the left	168	7.7	(6.6, 8.9)
The tail is usually carried to the right	140	6.4	(5.4, 7.5)
When lunging, which rein does your horse move more easily on? (LUNGE_PREFERENCE)			1815
Equal on both	358	19.7	(17.9, 21.6)
Left rein	866	47.7	(45.4, 50.0)
Right rein	591	32.6	(30.4, 34.8)
When riding, to which side does your horse bend more easily? (BEND_EASIER)			2253
Neither-both are equal	151	6.7	(5.7, 7.8)
Significantly more to the left	333	14.8	(13.3, 16.3)
Significantly more to the right	276	12.3	(10.9, 13.7)
Slightly more to the left	834	37.0	(35.0, 39.0)
Slightly more to the right	659	29.2	(27.4, 31.2)
Which direction do your horse’s hindquarters deviate towards when riding in a straight line?			
In walk (HINDQUARTER_DEVIATION_WALK)			1959
Deviate to the Left	425	21.7	(19.9, 23.6)
Deviate to the Right	273	13.9	(12.4, 15.5)
Neither	1261	64.4	(62.2, 66.5)
In trot (HINDQUARTER_DEVIATION_TROT)			1933
Deviate to the Left	466	24.1	(22.2, 26.1)
Deviate to the Right	334	17.3	(15.6, 19.0)
Neither	1133	58.6	(56.4, 60.8)
In left canter (HINDQUARTER_DEVIATION_CANTER_LEFT)			1743
Deviate to the Left	748	42.9	(40.6, 45.3)
Deviate to the Right	275	15.8	(14.1, 17.6)
Neither	720	41.3	(39.0, 43.7)
In right canter (HINDQUARTER_DEVIATION_CANTER_RIGHT)			1728
Deviate to the Left	318	18.4	(16.6, 20.3)
Deviate to the Right	661	38.3	(36.0, 40.6)
Neither	749	43.3	(41.0, 45.7)
When riding a trot-canter transition does your horse pick up/strike off on one canter lead more easily than the other? If so, which lead does the horse prefer? (CANTER_TRANSITION)			2208
Left lead	775	35.1	(33.1, 37.1)
Neither-they are the same	844	38.2	(36.2, 40.3)
Right lead	589	26.7	(24.8, 28.6)
In a leg yield, does your horse move more easily away from one leg? (EASY_MOVE)			2094
No, Left & Right are equal	474	22.6	(20.9, 24.5)
Yes, my Left leg	782	37.3	(35.3, 39.5)
Yes, my Right leg	838	40.0	(37.9, 42.2)
When riding your horse, does your saddle slip towards one side? (SADDLE_SLIP)			2244
No, the saddle remains Straight	1534	68.4	(66.4, 70.3)
Yes, it slips both Left and Right	50	2.2	(1.7, 2.9)
Yes, the saddle slips to the Left	274	12.2	(10.9, 13.6)
Yes, the saddle slips to the Right	386	17.2	(15.7, 18.8)
If your horse knows how to perform the half steps or piaffe, does the right or left limb take a longer step?			
Forelimbs (PIAFFE_FORE)			355
Both steps equal	183	51.5	(46.2, 56.9)
Left steps further forward than right	90	25.4	(20.9, 30.2)
Right steps further forward than left	82	23.1	(18.8, 27.8)
Hind limbs (PIAFFE_HIND)			352
Both steps equal	114	32.4	(27.5, 37.5)
Left steps further forward than right	133	37.8	(32.7, 43.1)
Right steps further forward than left	105	29.8	(25.1, 34.9)

**Table 3:** Cross-tabulated responses for questions if the horse’s shoulders fall in or out in left and in right direction while lunging (question 18) and while riding (question 24), and if the rider had more contact on the inside or outside rein in left and in right circles (question 22). Counts (n) and percentages with 95% confidence intervals (95% CI) are shown for each response option combination. Total n is shown in the rightmost column.

Questions/Response Options		n	%	95% CI
When lunging, does your horse do any of the following? (LUNGE_L/LUNGE_R)				
Left circle	Right circle			1788
Falls in	Falls out	208	11.6	(10.2, 13.2)
Falls in	Neither	213	11.9	(10.4, 13.5)
Falls in	Falls in	130	7.3	(6.1, 8.6)
Neither	Falls out	85	4.8	(3.8, 5.8)
Neither	Neither	485	27.1	(25.1, 29.3)
Neither	Falls in	366	20.5	(18.6, 22.4)
Falls out	Falls out	39	2.2	(1.6, 3.0)
Falls out	Neither	55	3.1	(2.3, 4.0)
Falls out	Falls in	207	11.6	(10.1, 13.2)
When ridden on a circle or a turn, on which rein does your horse’s shoulders fall out/fall in more easily? (SHOULDER_FALL_L/SHOULDER_FALL_R)				
Left circle	Right circle			2096
Falls in	Falls out	422	20.1	(18.4, 21.9)
Falls in	Neither	186	8.9	(7.7, 10.2)
Falls in	Falls in	79	3.8	(3.0, 4.7)
Neither	Falls out	181	8.6	(7.5, 9.9)
Neither	Neither	211	10.1	(8.8, 11.4)
Neither	Falls in	208	9.9	(8.7, 11.3)
Falls out	Falls out	112	5.3	(4.4, 6.4)
Falls out	Neither	192	9.2	(8.0, 10.5)
Falls out	Falls in	505	24.1	(22.3, 26.0)
When riding on circle, which side has more rein contact? (CONTACT_GREATER_L/CONTACT_GREATER_R)				
Left circle	Right circle			2277
Equal contact	More outside contact	99	4.3	(3.5, 5.3)
Equal contact	More inside contact	93	4.1	(3.3, 5.0)
Equal contact	Equal contact	389	17.1	(15.6, 18.7)
Equal contact	Inconsistent contact	16	0.7	(0.4, 1.1)
Inconsistent contact	Equal contact	12	0.5	(0.3, 0.9)
Inconsistent contact	Inconsistent contact	290	12.7	(11.4, 14.2)
Inconsistent contact	More outside contact	42	1.8	(1.3, 2.5)
Inconsistent contact	More inside contact	32	1.4	(1.0, 2.0)
More inside contact	Equal contact	88	3.9	(3.1, 4.7)
More inside contact	Inconsistent contact	20	0.9	(0.5, 1.4)
More inside contact	More outside contact	323	14.2	(12.8, 15.7)
More inside contact	More inside contact	89	3.9	(3.2, 4.8)
More outside contact	Equal contact	117	5.1	(4.3, 6.1)
More outside contact	Inconsistent contact	47	2.1	(1.5, 2.7)
More outside contact	More outside contact	306	13.4	(12.1, 14.9)
More outside contact	More inside contact	314	13.8	(12.4, 15.3)

**Table 4:** Variable loadings from multiple correspondence analysis of rider laterality variables (model A–all questions in Table 1), each variable represents a response option within a question. The capitalised part of each variable name is an acronym (see Table 1) indicating which question the response option belongs to. Data from 2299 questionnaires, the third column shows the count (n) per response option. The 3 dimensions (Dim) that explained the largest proportion of the total inertia are included, showing the 5 variables with the highest positive (1 to 5) and negative (−1 to −5) loadings, respectively.

Dim	Rank	n	Variable	Loading	End
1	1	65	WRITINGHAND Ambidextrous (I use both; It varies)	2.4	High
	2	169	BALLKICK Ambidextrous/Ambipedal (I use both; It varies)	2.0	High
	3	112	ARMFOLD Ambidextrous (I use both; It varies)	1.9	High
	4	256	CHAIRSTEP Ambidextrous/Ambipedal (I use both; It varies)	1.6	High
	5	28	MOUNTING Always from the Right Side	1.1	High
	−5	1461	MOUNTING Always from the Left Side	−0.4	Low
	−4	1295	CHAIRSTEP Right	−0.4	Low
	−3	16	HANDLING Always from the Right Side	−0.4	Low
	−2	353	LATERALITY No	−0.5	Low
	−1	353	HANDLING Always from the Left Side	−0.6	Low
2	1	176	BALLKICK Left	2.7	High
	2	266	WRITINGHAND Left	2.2	High
	3	748	CHAIRSTEP Left	0.8	High
	4	353	HANDLING Always from the Left Side	0.5	High
	5	1110	ARMFOLD Left	0.2	High
	−5	240	MOUNTING Both Sides	−0.4	Low
	−4	256	CHAIRSTEP Ambidextrous/Ambipedal (I use both; It varies)	−0.5	Low
	−3	16	HANDLING Always from the Right Side	−0.5	Low
	−2	28	MOUNTING Always from the Right Side	−0.5	Low
	−1	59	HANDLING Mostly from the Right Side	−0.6	Low
3	1	28	MOUNTING Always from the Right Side	5.6	High
	2	59	HANDLING Mostly from the Right Side	4.7	High
	3	28	MOUNTING Mostly from the Right Side	4.5	High
	4	16	HANDLING Always from the Right Side	2.8	High
	5	176	BALLKICK Left	0.4	High
	−5	857	HANDLING Both Sides	−0.2	Low
	−4	542	MOUNTING Mostly from the Left Side	−0.3	Low
	−3	240	MOUNTING Both Sides	−0.3	Low
	−2	169	BALLKICK Ambidextrous/Ambipedal (I use both; It varies)	−0.3	Low
	−1	65	WRITINGHAND Ambidextrous (I use both; It varies)	−0.9	Low

**Table 5:** Variable loadings from multiple correspondence analysis of horse and rider asymmetry/ side preference variables (model B-all questions in Table 1, Table 2 and Table 3 plus the lameness history questions), each variable represents a response option within a question. The capitalised part of each variable name is an acronym (see Table 1 and Table 2) indicating which question the response option belongs. Data from 2132 questionnaires, the third column shows counts (n) per response option. The 5 dimensions (Dim) that explained the largest proportion of the total inertia are included, showing the 10 variables with the highest positive (1 to 10) and negative (−1 to −10) loadings, respectively.

Dim	Rank	n	Variable	Loading
1	1	57	SADDLE_SLIP I’m not sure	1.33
	2	141	SHOULDER_FALL_R Not sure	1.27
	3	75	LUNGE_L Not sure	1.23
	4	75	LUNGE_R Not sure	1.20
	5	128	SHOULDER_FALL_L Not sure	1.19
	6	330	HINDQUARTER_DEVIATION_TROT Not sure	1.14
	7	305	HINDQUARTER_DEVIATION_WALK Not sure	1.13
	8	47	BEND_EASIER I’m not sure	1.11
	9	163	HINDQUARTER_MUSCLE I’m not sure	1.04
	10	103	TAIL_CARRIAGE I’m not sure	1.02
	−10	567	CONTACT_GREATER_R EQUAL rein contact	−0.59
	−9	555	SHOULDER_FALL_L Neither	−0.60
	−8	553	SHOULDER_FALL_R Neither	−0.61
	−7	436	EASY_MOVE No, Left & Right are equal	−0.61
	−6	699	HINDQUARTER_DEVIATION_CANTER_RIGHT Neither	−0.69
	−5	669	HINDQUARTER_DEVIATION_CANTER_LEFT Neither	−0.72
	−4	339	LUNGE_PREFERENCE Equal on both	−0.73
	−3	138	BEND_EASIER Neither-both are equal	−1.15
	−2	170	PIAFFE_FORE Both steps equal	−1.19
	−1	106	PIAFFE_HIND Both steps equal	−1.43
2	1	79	PIAFFE_FORE Right steps further forward than left	1.17
	2	122	PIAFFE_HIND Left steps further forward than right	1.02
	3	78	PIAFFE_FORE Left steps further forward than right	1.02
	4	381	LAME_RIGHT_DIAGONAL Yes	0.89
	5	99	PIAFFE_HIND Right steps further forward than left	0.89
	6	405	LAME_LEFT_LATERAL Yes	0.88
	7	412	LAME_LEFT_DIAGONAL Yes	0.84
	8	415	LAME_RIGHT_LATERAL Yes	0.82
	9	725	LAME Yes	0.71
	10	187	HINDQUARTER_MUSCLE The right hind has increased muscle development	0.66
	−10	122	EASY_MOVE Not applicable-I don’t do leg yield	−0.87
	−9	342	LUNGE_R Don’t Lunge	−0.89
	−8	339	LUNGE_L Don’t Lunge	−0.89
	−7	339	LUNGE_PREFERENCE Not applicable-I don’t lunge	−0.89
	−6	305	HINDQUARTER_DEVIATION_WALK Not sure	−1.03
	−5	330	HINDQUARTER_DEVIATION_TROT Not sure	−1.03
	−4	87	CANTER_TRANSITION Not sure	−1.14
	−3	141	SHOULDER_FALL_R Not sure	−1.48
	−2	128	SHOULDER_FALL_L Not sure	−1.62
	−1	47	BEND_EASIER I’m not sure	−1.75
3	1	415	LAME_RIGHT_LATERAL Yes	1.24
	2	412	LAME_LEFT_DIAGONAL Yes	1.21
	3	381	LAME_RIGHT_DIAGONAL Yes	1.17
	4	405	LAME_LEFT_LATERAL Yes	1.15
	5	339	LUNGE_L Don’t Lunge	1.03
	6	342	LUNGE_R Don’t Lunge	1.02
	7	339	LUNGE_PREFERENCE Not applicable-I don’t lunge	0.99
	8	725	LAME Yes	0.87
	9	138	BEND_EASIER Neither-both are equal	0.85
	10	26	MOUNTING Always from the Right Side	0.59
	−10	551	LUNGE_PREFERENCE Right rein	−0.37
	−9	537	LUNGE_L Falls In	−0.37
	−8	402	HINDQUARTER_DEVIATION_WALK Deviate to the Left	−0.38
	−7	163	HINDQUARTER_MUSCLE I’m not sure	−0.39
	−6	178	FRONT_HOOVES I’m not sure	−0.43
	−5	1407	LAME No	−0.45
	−4	316	LUNGE_R Falls Out	−0.46
	−3	27	MOUNTING Mostly from the Right Side	−0.47
	−2	103	TAIL_CARRIAGE I’m not sure	−0.52
	−1	99	LUNGE_PREFERENCE I’m not sure	−0.54
4	1	339	LUNGE_L Don’t Lunge	1.77
	2	342	LUNGE_R Don’t Lunge	1.76
	3	339	LUNGE_PREFERENCE Not applicable-I don’t lunge	1.68
	4	308	HINDQUARTER_DEVIATION_TROT Deviate to the Right	0.56
	5	14	HANDLING Always from the Right Side	0.55
	6	99	PIAFFE_HIND Right steps further forward than left	0.55
	7	254	HINDQUARTER_DEVIATION_WALK Deviate to the Right	0.54
	8	27	MOUNTING Mostly from the Right Side	0.51
	9	257	HINDQUARTER_DEVIATION_CANTER_LEFT Deviate to the Right	0.40
	10	187	HINDQUARTER_MUSCLE The right hind has increased muscle development	0.39
	−10	138	BEND_EASIER Neither-both are equal	−0.48
	−9	87	CANTER_TRANSITION Not sure	−0.50
	−8	889	LUNGE_L Neither	−0.50
	−7	714	LUNGE_R Neither	−0.56
	−6	141	SHOULDER_FALL_R Not sure	−0.62
	−5	128	SHOULDER_FALL_L Not sure	−0.66
	−4	75	LUNGE_L Not sure	−0.70
	−3	339	LUNGE_PREFERENCE Equal on both	−0.74
	−2	75	LUNGE_R Not sure	−0.76
	−1	47	BEND_EASIER I’m not sure	−0.88
5	1	27	MOUNTING Mostly from the Right Side	1.25
	2	257	BEND_EASIER Significantly more to the right	0.87
	3	55	HANDLING Mostly from the Right Side	0.85
	4	551	LUNGE_PREFERENCE Right rein	0.84
	5	673	SHOULDER_FALL_L Falls In	0.80
	6	537	LUNGE_L Falls In	0.71
	7	533	CANTER_TRANSITION Right lead	0.67
	8	316	LUNGE_R Falls Out	0.57
	9	75	LUNGE_R Not sure	0.56
	10	609	BEND_EASIER Slightly more to the right	0.53
	−10	776	SHOULDER_FALL_L Falls Out	−0.40
	−9	555	SHOULDER_FALL_L Neither	−0.41
	−8	292	LUNGE_L Falls Out	−0.43
	−7	768	BEND_EASIER Slightly more to the left	−0.44
	−6	500	CONTACT_GREATER_R Higher rein contact on the INSIDE rein	−0.51
	−5	716	CANTER_TRANSITION Left lead	−0.51
	−4	804	LUNGE_PREFERENCE Left rein	−0.68
	−3	752	SHOULDER_FALL_R Falls In	−0.74
	−2	685	LUNGE_R Falls In	−0.75
	−1	313	BEND_EASIER Significantly more to the left	−0.77

## Data Availability

Dataset available on request from the authors.

## Supplementary Materials

The following supporting information can be downloaded at: https://www.mdpi.com/article/10.3390/ani15131956/s1, Supplement S1. The English questionnaire shared online. Supplement S2.: Table S1: Country distribution of those responding to an internet questionnaire about sidedness of horses administered 22 November 2022 – 22 March 2023. Table S2: Breeds of the horses in the sample (n=2304). Answers were translated to English and free text answers were post-categorised to a large degree. Breeds beginning with capital letters are post-categorised, while lower case breed are presented as found. Data from an internet questionnaire about sidedness of horses administered 22 November 2022 – 22 March 2023. Table S3: Colours of the horses in the sample (n=2304). Answers were translated to English and free text answers were post-categorised to a large degree. Data from an internet questionnaire about sidedness of horses administered 22 November 2022–22 March 2023. Table S4: Numbers, percentages and 95% confidence intervals (95% CI) for questions and response alternatives related to horse sidedness, including also ‘Im not sure- not sure’ answers or ‘not applicable’ answers. Data from an internet questionnaire about sidedness of horses administered 22 November 2022–22 March 2023.
